# Supplementary material for: Psychometric Evaluation of Women’s Knowledge of Healthcare Rights and Perception of Resource Scarcity during Maternity
Source: Healthcare (Basel). 2024 Oct 15;12(20):2045. doi: 10.3390/healthcare12202045 (PMC11508016; doi:10.3390/healthcare12202045)
Supplement: Supplementary file 1 [file healthcare-12-02045-s001.zip › healthcare-3213223-supplementary.pdf]

Supplementary material

Table S1. Expert´s scores, content validity index and Aiken's V coefficient in each item and global for MatCODE and MatER questionaries. CVI-i: Content validity index item; SEM: standard error of mean. Aiken's V coefficient was calculated considering the language clarity, relevance, and coherence domains.

MatCODE	Expert 1	Expert 2	Expert 3	Expert 4	Expert 5	CVI-i	Aiken's V
Item 1	7	13	15	15	15	0.87	0.83
Item 2	11	14	11	13	14	0.84	0.80
Item 3	14	15	15	15	15	0.99	0.98
Item 4	15	15	15	15	15	1.00	1.00
Item 5	15	14	15	15	15	0.99	0.98
Item 6	9	15	15	15	14	0.91	0.88
Item 7	15	15	15	15	15	1.00	1.00
Item 8	15	15	15	15	14	0.99	0.98
Item 9	15	15	15	15	15	1.00	1.00
Item 10	15	15	7	15	14	0.88	1.00
Item 11	14	15	7	15	15	0.88	1.00
Mean±SEM	13.2±0.86	14.6±0.20	13.2±0.99	14.8±0.18	14.6±0.15	0.94±0.02	0.96±0.02
**MatER**	**Expert 1**	**Expert 2**	**Expert 3**	**Expert 4**	**Expert 5**	**CVI-i**	**Aiken's V**
Item 1	15	15	15	15	15	1.00	1.00
Item 2	14	15	15	15	15	0.99	0.98
Item 3	12	15	15	15	15	0.96	1.00
Item 4	14	15	15	15	15	0.99	0.98
Item 5	14	15	15	13	15	0.96	0.95
Item 6	15	15	15	15	15	1.00	1.00
Item 7	15	15	15	15	15	1.00	1.00
Item 8	15	15	15	15	15	1.00	1.00
Item 9	15	15	15	15	15	1.00	1.00
Mean±SEM	14.3±0.33	15.0±0.00	15.0±0.00	14.8±0.22	15.0±0.00	0.99±0.01	0.99±0.01

Figure S1. Pearson´s correlation coefficients of the items from the questionnaires focus on women's knowledge of healthcare rights (MatCODE) and perception of resource scarcity (MatER) during pregnancy, labor and early postpartum.

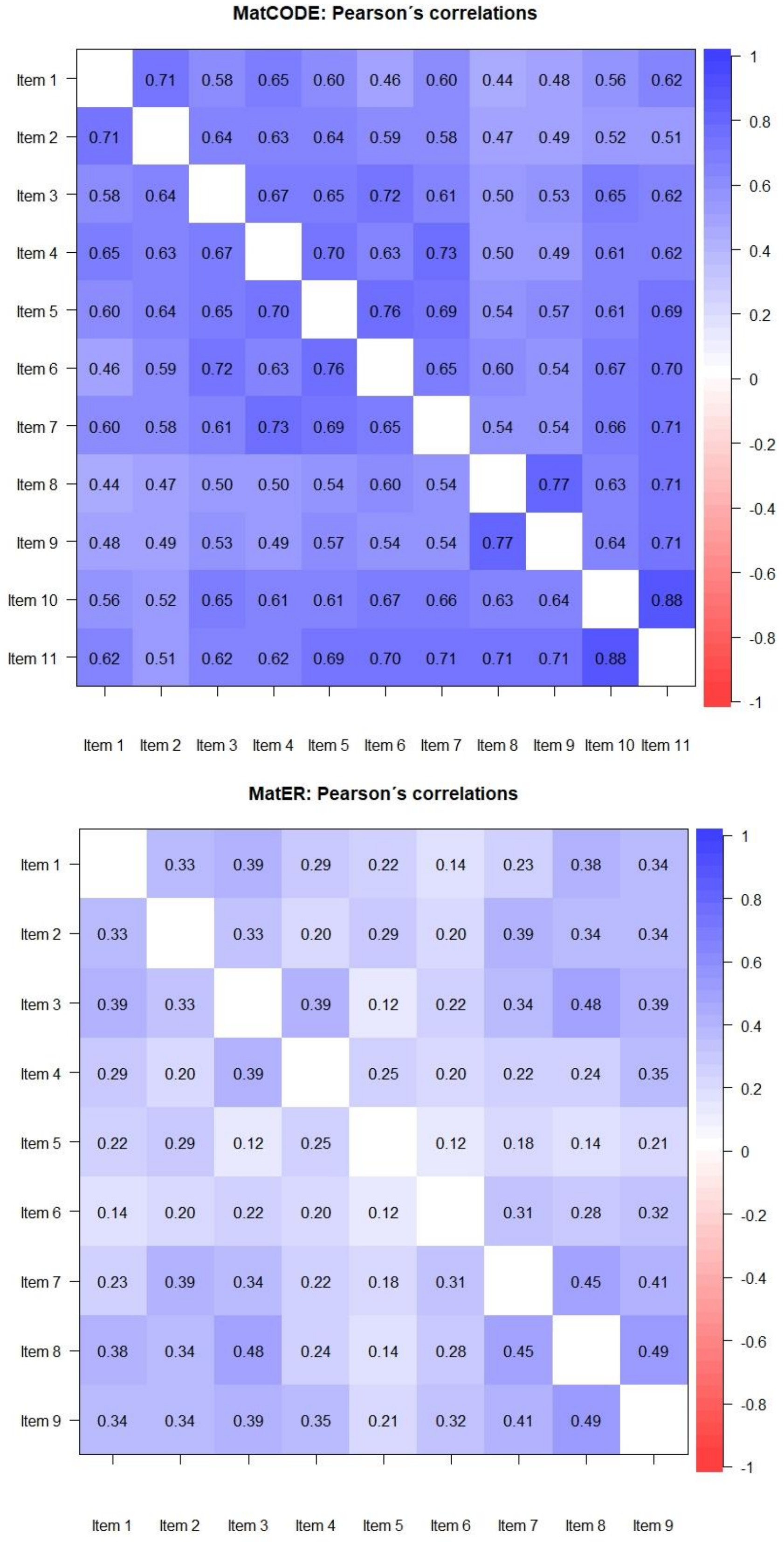

Table S2. The divergent analysis removing item 5 from MatER. Pearson´s correlation coefficient regarding to MatER scores with validated psychometric tests. RS-14: the Resilience scale; PANAS+: the Positive and Negative Affect Schedule positive score; PANAS-: the Positive and Negative Affect Schedule negative score; MBS-life: the Maternity Beliefs Scale maternity as a sense of life domain; MBS-social: the Maternity Beliefs Scale as a social duty domain. A p-Value (P)<0.05 was considered statistically significant.

	**MatER**
RS-14	-0.34 [-0.48; -0.19] P<0.001
PANAS+	-0.46 [-0.58; -0.31] P<0.001
PANAS-	0.49 [0.36; 0.61] P<0.001
MBS-life	0.01 [-0.16; 0.17] P=0.940
MBS-social	0.03 [-0.13; 0.20] P=0.685

Table S3. The known-groups validation removing item 5 from MatER. Data shown mean±standard error of mean (SEM). Statistically significant was established as p-value (P)≤0.05 by Mann–Whitney U-test.

		Mean±SEM	P			Mean±SEM	P
Parity	Primiparous	10.13±0.69	0.322	Last labor	C-section	10.10±0.75	0.372
	Multiparous	9.17±0.75			Vaginal	9.38±0.69	
Civil status	Single/unmarried	11.12±1.61	0.543	Pregnancy complications	Yes	10.93±0.96	0.109
	In relationship	9.37±0.51			No	9.11±0.96	
Work situation	Unemployed	10.52±0.80	0.130	Labor complications	Yes	11.11±1.23	0.123
	Active working	8.97±0.64			No	9.35±0.56	
Planned pregnancy	Yes	8.41±0.61	0.022	Postpartum complications	Yes	13.23±1.26	0.003
	No	11.13±0.81			No	9.04±0.54	
Desired pregnancy	Yes	9.06±0.51	0.009				
	No	13.79±1.69					
